# A Rare Case of Right Renal Vein Entrapment: A Cadaveric Study

**DOI:** 10.7759/cureus.70507

**Published:** 2024-09-30

**Authors:** Audrey Bourdages, Alyssa Breazeale, Rachel Gatewood, Cade Hunter, Daniella Patel, Alexis Salters, Uzochukwu Adabanya, Matthew D Overturf

**Affiliations:** 1 Medicine, Edward Via College of Osteopathic Medicine, Monroe, USA; 2 Anatomical Sciences, Edward Via College of Osteopathic Medicine, Monroe, USA

**Keywords:** cadaver case report, middle suprarenal artery, nutcracker syndrome (ncs), rare anatomical variants, right renal vein compression

## Abstract

Renal vein entrapment, especially concerning the right renal vein, represents a scarcely explored anatomical aberration. The right renal vein's pivotal role in renovascular renal hemodynamics underlines the clinical significance of its compression, which can precipitate an elevated renal venous pressure gradient in relation to the inferior vena cava. This report delineates a unique instance of right renal vein entrapment in a 92-year-old male cadaver, identified during routine dissection. This entrapment is due to an unusual course of the right middle suprarenal artery that originates from the abdominal aorta and traverses inferior and retrocaval, causing the middle suprarenal artery to course anteriorly and superiorly to the right renal vein. This case, not paralleled in the extant literature, bears resemblance to the left renal vein entrapment in nutcracker syndrome (NCS) and thereby raises conjectures about possible renal manifestations akin to NCS in similar anatomical anomalies. The primary objective of this report is to augment the understanding and clinical relevance of this rare anatomical deviation in renal health.

## Introduction

Entrapment of the left renal vein is associated with nutcracker syndrome (NCS) due to the origin of the left testicular vein; however, the implications of compressing the right renal vein are not well documented. NCS is a relatively rare condition that occurs when there is compression of the left renal vein, most commonly between the superior mesenteric artery and the aorta [1]. This results in the left testicular vein retaining blood due to redistribution of the volume that is no longer able to drain through the left renal vein. NCS in men may present as varicoceles in addition to flank pain and hematuria with possible proteinuria [1]. NCS is associated with infertility and, if left untreated, may contribute to morbidities such as chronic renal disease and venous thrombosis [1]. The middle suprarenal artery has been documented as having varying origins but lacks documentation of instances in which the right middle suprarenal artery wraps around the right renal vein resulting in proximal dilation. This case report will evaluate the effect of compression and subsequent dilation of the right renal vein caused by the course of the compression of the right middle suprarenal artery as well as implications in treatment.

## Case presentation

**Figure 1:**
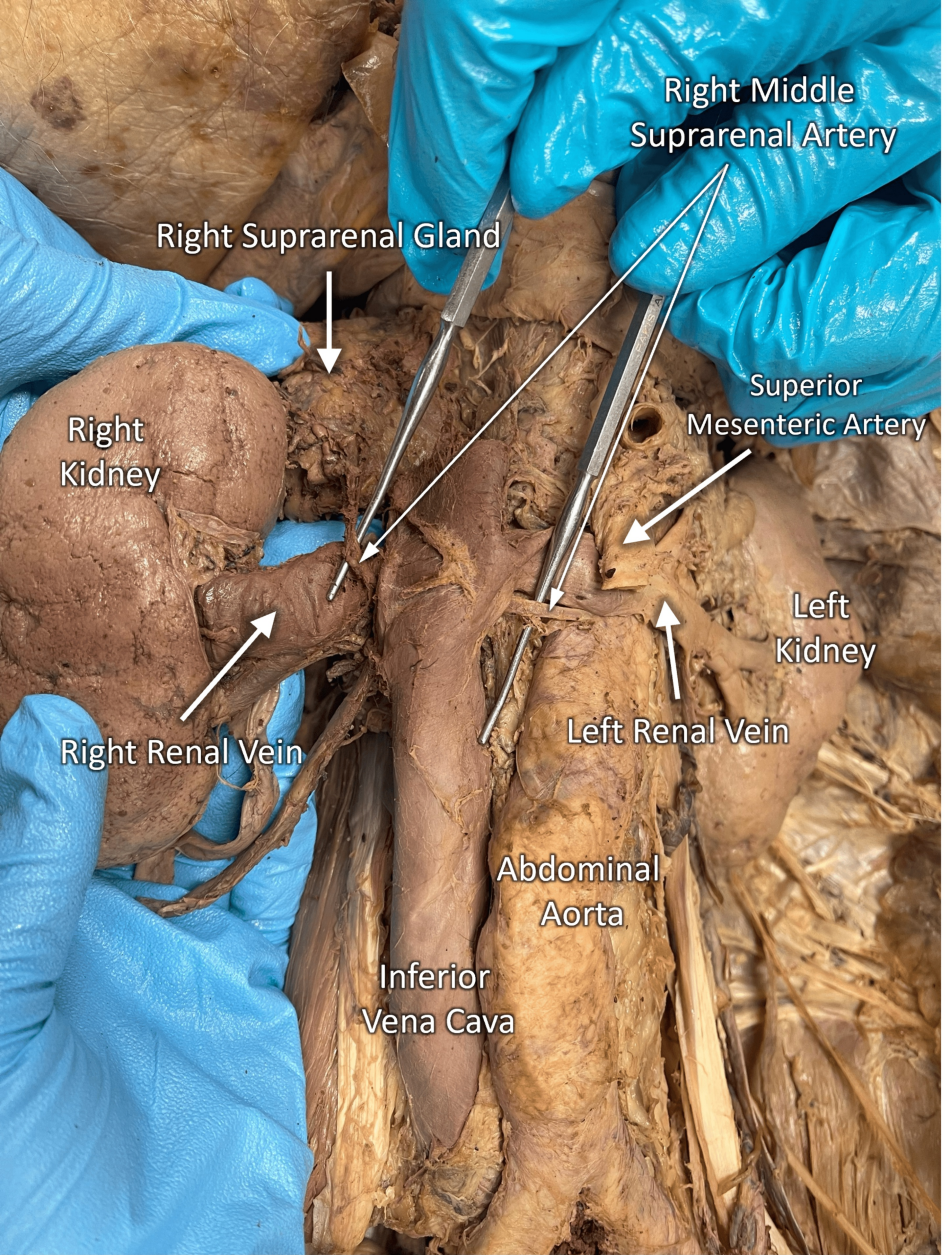
Right renal vein entrapment Presentation of right renal vein entrapment due to the course of the right middle suprarenal artery in the cadaver of a 92-year-old Caucasian male.​

During the cadaveric dissection of a 92-year-old Caucasian male with a history of hepatocellular carcinoma, we observed the entrapment of the right renal vein. This was due to the right middle suprarenal artery taking a rare arbitrary path following its origination from the abdominal aorta. The right middle suprarenal artery coursed inferiorly and retrocaval, requiring it to pass superiorly over the right renal vein to supply the adrenal (suprarenal) gland. This anatomical anomaly resulted in compression and relative dilation of the right renal vein (Figure 1). As expected, the right renal artery coursed posteriorly to the inferior vena cava and right renal vein before entering the hilum of the right kidney. The right superior and inferior suprarenal arteries followed a normal course, neither of which contributed to the compression of the right renal vein. No anatomical variations in the vasculature of the left kidney were observed.

## Discussion

NCS is classically defined as the entrapment of the left renal vein, and this typically occurs between the superior mesenteric artery and the aorta as the left renal vein courses from the left kidney to the inferior vena cava. The syndrome's epidemiology is nebulous as the diagnostic criteria and variability in presentations have detracted from consistent surveillance. That said, NCS is at present considered an uncommon clinical disorder. Peak prevalence is suggested to occur between the third and fourth decades of life [2]. Our subject's symptoms are exceedingly rare as right renal vein entrapment, or inverted NCS, is the primary indication for this case study. Right renal vein entrapment is characterized by impeded outflow from the right renal vein into the inferior vena cava. In the present case, entrapment is due to the path of the right middle suprarenal artery. Right renal vein entrapment has been documented twice in English medical literature prior to this study. These cases were observed with a left-sided inferior vena cava with a hemiazygos vein continuation and persistent left superior vena cava [2,3]. No firm conclusions can be drawn at this time about the epidemiology of inverted NCS in comparison to the classical variant. 

Due to the presence of some physiological similarities between right renal vein and left renal vein entrapment, it is hypothesized that the patient would have experienced certain symptoms like those present in patients with NCS. In NCS, patients' presentations vary from asymptomatic microhematuria to severe pelvic congestion [4]. Diagnosis of NCS is supported by a clinical assemblage of intractable flank or pelvic pain accompanied by microscopic or macroscopic hematuria and proteinuria. Hematuria is the result of venous hypertension which can lead to the rupture of thin-walled veins into the collecting system [4]. Renal vein entrapment and subsequent increased renal venous pressure can lead to the development of proteinuria. Renal vein entrapment can reduce renal blood flow which causes a reduction in the glomerular filtration rate (GFR) inducing the release of angiotensin II that works to constrict the renal efferent arteriole to maintain the GFR in the desired physiological range [5,6]. The increase in GFR leads to proteinuria. Additional symptoms seen in classical NCS such as testicular varicoceles and pelvic congestion would likely be absent as the right testicular and ovarian vein drain directly into the inferior vena cava [7,8].

The primary step in the diagnosis of right renal vein entrapment is a thorough physical examination and patient history. The findings of the examination and history would parallel common symptoms and functional impairments that are present in NCS. Once right renal vein entrapment is suspected, Doppler ultrasound should be performed as it is noninvasive, inexpensive, and can aid in diagnosis as it provides details regarding the physical characteristics (e.g. relative size differences, reduced blood flow) of the renal vessels. For NCS diagnosis, a diagnostic criterion facilitated via ultrasound consists of standard ultrasonographic findings, positional changes, and urinalysis [9]. Depending on the clinical scenario, additional diagnostic methods may be utilized to rule out more common renal conditions and to further confirm suspicion. These include, but are not limited to, blood examinations, urinalysis, urine culture, cytology, urethrocystoscopy, CT urography, renal biopsy, renal angiography, angiographic CT, digital subtraction angiography, standard magnetic resonance (MR) imaging, and MR angiography [4].

Treatment, just like in NCS, should be based on the severity of symptoms and the potential of the condition to reverse based on age and stage of disease. Treatment options can include observation, medical therapy, or surgery. In younger patients, conservative treatment with an observational approach is preferred in NCS due to the high probability of reversal of hematuria. Angiotensin-converting enzyme inhibitors and angiotensin receptor blockers may be used to help decrease proteinuria by decreasing the effects of angiotensin II on the efferent arterioles [4].

Once symptoms become severe, we can consider a surgical approach to lower the right renal venous hypertension. This may involve bypasses, shunts, transposition, stents, grafts, nephrectomy, and renal transplant. Selection criteria for these procedures are not well-defined in NCS and are based on the patient's severity and presentation; therefore, they must be approached in the same manner in right renal vein entrapment [4].

## Conclusions

The purpose of this case is to document the unique circumstance of right renal vein entrapment caused by the compressive forces of the right middle suprarenal artery and the right renal artery, a rare anatomical variant that is not well documented. Given the lack of documentation in the literature, it is difficult to definitively conclude how a patient with this condition would present; however, one can postulate that symptoms could include some of those seen in patients with NCS. These include pelvic/flank pain, hematuria, and proteinuria. Asymptomatic individuals may not need treatment; however, those who experience recurrent symptoms such as pain, proteinuria, and hematuria due to renal vein entrapment require medical therapy, with refractory cases necessitating surgical intervention.
